# The nucleotide addition cycle of RNA polymerase is controlled by two molecular hinges in the Bridge Helix domain

**DOI:** 10.1186/1741-7007-8-134

**Published:** 2010-10-29

**Authors:** Robert OJ Weinzierl

**Affiliations:** 1 Department of Life Sciences, Imperial College London, London SW7 2AZ, UK

## Abstract

**Background:**

Cellular RNA polymerases (RNAPs) are complex molecular machines that combine catalysis with concerted conformational changes in the active center. Previous work showed that kinking of a hinge region near the C-terminus of the Bridge Helix (BH-H_C_) plays a critical role in controlling the catalytic rate.

**Results:**

Here, new evidence for the existence of an additional hinge region in the amino-terminal portion of the Bridge Helix domain (BH-H_N_) is presented. The nanomechanical properties of BH-H_N _emerge as a direct consequence of the highly conserved primary amino acid sequence. Mutations that are predicted to influence its flexibility cause corresponding changes in the rate of the nucleotide addition cycle (NAC). BH-H_N _displays functional properties that are distinct from BH-H_C_, suggesting that conformational changes in the Bridge Helix control the NAC via two independent mechanisms.

**Conclusions:**

The properties of two distinct molecular hinges in the Bridge Helix of RNAP determine the functional contribution of this domain to key stages of the NAC by coordinating conformational changes in surrounding domains.

## Background

RNA polymerases (RNAPs) play a central role in the regulation of gene expression. Like the majority of the enzymes involved in fundamental biological information-processing functions (for example, replication, transcription, recombination, repair), RNAPs are probably best viewed as intricate molecular machines. The movement of nucleic acid substrates, coupled with various types of active site chemistries, requires a precisely orchestrated sequence of conformational changes of protein domains during the transcription cycle (for recent reviews see [1-4]).

The nanomechanical mechanisms guiding the structural rearrangements of domains within the active site are still very poorly understood. Thus far, models of the fundamental reaction catalyzed by RNAPs, the nucleotide addition cycle (NAC), have predominantly been derived from a series of crystal structures that contain RNAPs as apoenzymes (for example [5-9]), or complexed with various substrates and inhibitors (for example [10-15]). Such structures, revealing (among other features) pre- and post-translocation states of RNAPs, have provided the basis for various hypotheses concerning the molecular mechanism of the NAC [1-4,16,17]. There are, however, two potential shortcomings associated with such approaches. First, in order to 'freeze' the RNAPs in a crystallizable conformation, substrate analogs or inhibitors need to be chosen that stop the reaction cycle at a specific point. This may result in the adoption of 'off-pathway' conformations that do not represent normal enzyme states. A second, more fundamental, problem is that short-lived intermediate structures cannot be captured in crystals because they are thermodynamically or kinetically unstable. Yet, it is likely that an awareness of the existence and functional significance of such intermediates will be required to develop a deeper understanding of the mechanisms operating within molecular machines.

**Figure 1:**
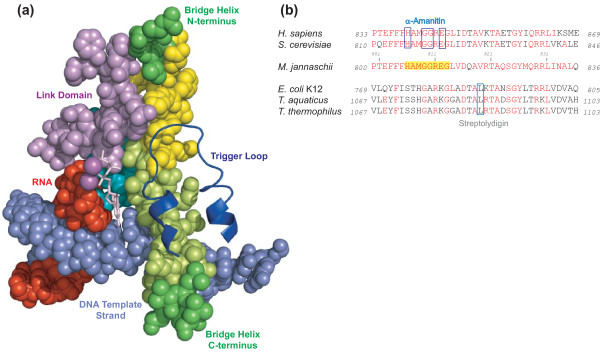
**Evolutionary conservation and arrangement of domains in the RNAP active site**. **(A) **Most structures are shown in space-filling mode to emphasize spatial connections. The Bridge Helix is shown in green, with the regions subjected to high-throughput mutagenesis in this study highlighted in yellow. The template DNA is pale blue, the RNA is red, the NTP in the insertion site is shown as a pink stick model and catalytic metal ions as magenta spheres. Three additional domains that surround the Bridge Helix N-terminus, β-D II domain (turquoise) and Link (light purple) and the Trigger Loop (dark blue cartoon) are shown. (PDB #2E2H). **(B) **Alignment of Bridge Helix sequences from bacteria (*Escherichia coli *K12 (Genbank BAE77332); *Thermus aquaticus *(Genbank RPOC_THEAQ); *Thermus thermophilus *(Genbank RPOC_THET8) and eukaryotes (*Saccharomyces cerevisiae *(Genbank CAA26904) and *Homo sapiens *(Genbank EAW90183)) against the archaeon *M. jannaschii *(Genbank A64430). Residues identical to the archaeal sequence are shown in red. The region mutagenized is highlighted with yellow background. The residues contacted by α-amanitin (eukaryotic RNAPII) and streptolydigin (bacterial RNAPs) are boxed. The numbers flanking the sequences represent the location of the sequences within the open reading frame of the complete subunit.

We have designed new experimental tools to complement ongoing structural investigations. Based on the ability to assemble an active RNAPII-like enzyme from recombinant subunits *in vitro*, it is possible to modify any residue within an intact RNAP to introduce a variety of targeted mutations into any functional domain participating in the NAC [18]. Such a strategy not only allows specific predictions based on available X-ray structures to be tested, but also can be used to explore systematically the functional contributions of individual domains to biochemically detectable activities. New robotic methods that facilitate the labor-intense high-throughput mutagenesis/assembly steps and transcription assays allow this approach to be implemented on a large scale [19]. In a recent study we systematically replaced each of 17 consecutive residues of the Bridge Helix domain with all other 19 possible amino acid side chains [20]. The Bridge Helix, a 35 amino acid α-helix spanning the RNAP active site, controls the flow of nucleic acid substrates and nucleotide precursors through the catalytic site (Figure 1A). Some of the earliest models of the NAC were based on the observation that the C-terminal portion of the Bridge Helix was kinked in some X-ray crystal structures of bacterial RNAPs [5,8], but appeared straight in numerous other crystals of bacterial, archaeal and eukaryotic RNAPs. The existence of two alternative Bridge Helix conformations made it seem likely that a periodic oscillation between straight and kinked conformations would be implicated in translocating RNAPs in single base-pair steps along the DNA template strand [7,10,21-24]. High-throughput mutagenesis of the C-terminal portion of the Bridge Helix provided unexpectedly clear evidence in support of Bridge Helix kinking; mutations destabilizing the normal α-helical conformation in certain positions cause a substantial increase in the specific activity of RNAPs. This phenomenon, referred to as superactivity, relieves a constraint on the catalytic mechanism by increasing the frequency of Bridge Helix isomerization between straight and kinked conformations [20,25].

While the combination of structural observations and mutagenesis data clearly highlights the functional contribution of the C-terminal portion of the Bridge Helix towards controlling the rate of the NAC, the role of the N-terminal portion of the Bridge Helix has thus far remained enigmatic. The primary sequence of this region is exceptionally highly conserved during evolution; for example, the sequences of the N-terminal 15 amino acids are identical between the archaeon *Methanocaldococcus jannaschii *and humans, and differ by only a single residue from yeast (Figure 1B). Such a high degree of structural conservation over more than two billion years of evolution can be partially accounted for by the fact that the Bridge Helix N-terminus is tightly surrounded by other domains and may therefore be spatially and evolutionarily constrained due to the need to maintain an extensive network of protein-protein interactions (Figure 1A; Additional files 1, 2 and 3; [12,15,25,26]). In apparent agreement with this view, all available X-ray structures of RNAPs show the N-terminal portion of the Bridge Helix in a rigidly α-helical conformation, suggesting the absence of significant conformational changes. For this reason, none of the current models of RNAP function consider the Bridge Helix N-terminus to play any dynamic role during the NAC [1-4,16,17].

New evidence presented here, based on a combination of high-throughput mutagenesis studies and molecular dynamics simulations, demonstrates that such a static view of the Bridge Helix N-terminus is untenable. The results show that this region contains a highly localized molecular hinge, and that the conformation of this site has a substantial influence on the rate of the NAC. In combination with the previously identified C-terminal hinge region, the data reinforces the overarching concept that the Bridge Helix plays a predominantly nanomechanical role during the translocation stage of the NAC by coordinating conformational changes in surrounding domains.

## Results

### High-throughput mutagenesis reveals evidence for an N-terminal hinge

The N-terminus of the Bridge Helix of the RNAP from the euryarchaeon *Methanocaldococcus jannaschii *was dissected by high-throughput mutagenesis [19,20]. In this automated approach, each amino acid within the target region (Figure 1B) was replaced with all other 19 residues to reveal local structural requirements. The mutants were then assayed in robotic promoter-independent transcription assays, which provide a consistent measure of the synthetic rate of the NAC and correlate directly with results obtained from a variety of promoter-dependent, abortive- and elongation transcription assays ([18-20]; S. Wiesler and ROJW, unpublished data).

**Figure 2:**
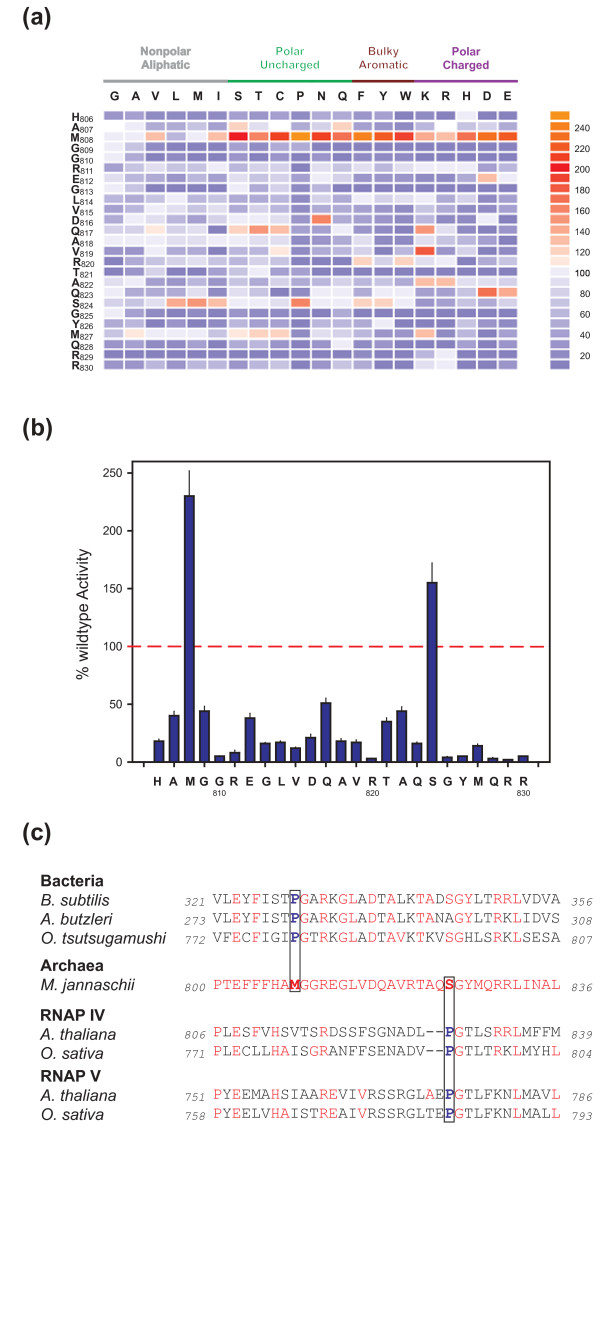
**High-throughput mutagenesis of the Bridge Helix**. **(A) **The specific activities in recruitment-independent transcription assays of systematic substitutions of archaeal Bridge Helix residues H806 to R830 are shown as a heat map relative to the activity of the wildtype enzyme (see adjacent scale for comparison). Substitutions in M808 cause an exceptional increase in catalytic activity with chemically diverse side chains. Previously published data (L814 to R830; [20]) are included to provide context. All assays were performed in at least quadruplicate with standard deviations within 12% of the average value. **(B) **Plot of proline substitutions across the Bridge Helix. The wildtype activity level (100%) is marked with a dashed red line. The substituted residues in the *M. jannaschii *Bridge Helix are shown along the horizontal axis. Most proline substitutions cause a severe reduction in the specific activity, except at positions M808 and S824, where proline substitutions cause superactivity. All assays were performed in at least quadruplicate, with error bars showing standard deviation from the average value. **(C) **Naturally occurring proline-substitutions (highlighted in boxes). The Bridge Helix sequences of three bacterial species, *Orientia tsutsugamushi *(Genbank YP_001248195 (Boryong)/YP_001938485 (Ikeda)), isolates of *Arcobacter butzleri *(Genbank AAZ80810) and *Bacillus subtilis *(Genbank BAA10999), as well as representative examples of plant RNAP IV and V Bridge Helix sequences from *Arabidopsis thaliana *(Genbank AAY89363 and NP_181532, respectively) and *Oryza sativa *(Genbank EEE70198 and EEE56320, respectively) are aligned against the *M. jannaschii *sequence (Genbank A64430). The bacterial sequences each contain a single proline residue corresponding to *mj*A' M808, whereas proline substitutions in RNAP IV and V align with *mj*A' S824. Residues identical to the archaeal sequence are shown in red.

The results reveal unexpected insights into the function of the Bridge Helix N-terminus (Figure 2A). The most eye-catching phenotypes are associated with the *mj*A' M808 position, which constitutes - both qualitatively and quantitatively - a hotspot for superactivity caused by a chemically diverse range of substitutions. This suggests that the M808 position is structurally very delicately balanced and substitutions with more hydrophilic, bulky hydrophobic, and/or charged residues cause a substantial local disturbance. The degree of superactivity in the strongest N-terminal substitution (*mj*A' M808-P; >240% superactivity) greatly exceeds the highest level of superactivity displayed by the strongest C-terminal mutant (*mj*A' S824-P; approximately 170% superactivity; Figure 2A). The observation that the replacement of M808 with proline results in the highest increase in specific activity immediately suggests that the phenotypes are caused predominantly by disruption of the local secondary structure; proline residues destabilize α-helices due to a lack of hydrogen bonding and steric interference with the backbone of the preceding turn [27]. In a manner highly reminiscent of the previously characterized *mj*A' S824-P substitution phenotype, proline substitutions of the residues immediately surrounding *mj*A' M808 cause a sharp drop in RNAP catalytic activity, emphasizing the highly localized effect of such conformationally induced changes (Figure 2B; [20]). The locations of two functionally acceptable proline substitutions thus mark the presence of two discrete and separate molecular hinges, that will subsequently be referred to as BH-H_N _('Bridge Helix - Hinge _N-terminal_'; typified by *mj*A' M808-P) and BH-H_C _('Bridge Helix - Hinge _C-terminal_'; typified by *mj*A' S824-P). The shared functional property of M808-P and S824-P substitutions, causing maximal levels of superactivity, emphasizes that increased Bridge Helix kinking at the two hinges correlates directly with an increased rate of nucleotide addition [20,25].

Extensive database searches, covering completed genome sequences of a large variety of pro- and eukaryotic species, show that naturally occurring proline substitutions in the Bridge Helix primary sequences are exceptionally rare. The only known instances of proline residues occurring naturally anywhere in the N-terminal part of the Bridge Helix are found in the bacteria *Orientia tsutsugamushi *[28,29], and certain isolates of *Arcobacter butzleri *[30] and *Bacillus subtilis *[31]. In each case, the substituted position is precisely orthologous to *mj*A' M808 (Figure 2C). In the C-terminal half of the Bridge Helix, the highly divergent plant RNAPIV and RNAPV enzymes display a strong tendency for a proline residue at the position orthologous to archaeal S824 (Figure 2C; [32,33]).

### Molecular dynamics reveal a structural basis for the BH-H_N _hinge mechanism

**Figure 3:**
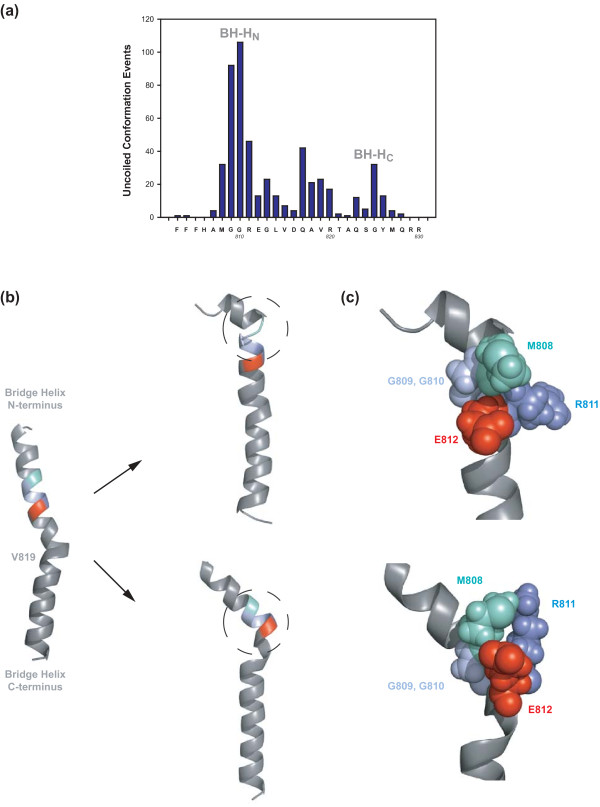
**Structural basis of BH-H_N _kinking**. **(A) **Overview of local unfolding events incurred by a *M. jannaschii *Bridge Helix model during 27 independent 200 picosecond MD simulations. The number of 5 picosecond windows during which a specific part of the Bridge Helix adopts a 'coil' conformation are plotted against the Bridge Helix sequence shown on the horizontal axis. A major area of α-helical instability, BH-H_N _(centered on *mj*A'G810), is evident from this semi-quantitative analysis. Additional unstable regions include BH-H_C _(centered on *mj*A' G825) and a 'labile region' (spanning *mj*A'Q817 to R820; see Discussion for more details). **(B) **Examples of kinked BH-H_N _conformations arising from MD simulations. The Bridge Helix on the left represents the starting conformation as modelled on the yeast RNAPII structure PDB #2E2H. The minor bulge near the center of the Bridge Helix corresponds to the V819 position. During unrestrained simulation, different types of kinked BH-H_N _structures (circled) involving *mj*A' G809/G810 (pale blue) and M808 (pale green), R811 (blue) and E812 (red) side-chain interaction emerge stochastically. **(C) **Structural details of BH-H_N _kinking models. The relevant residues are shown in space-filling mode to illustrate spatial relationships. Two glycine residues, *mj*A' G809 and G810 (pale blue) form a highly flexible hinge that allows M808 (pale green) to interact extensively via van der Waals interactions with the side-chains of R811 (blue) and E812 (red). In some cases these interactions create a stretched 3_10 _helix immediately C-terminal to R811 and E812.

Insights into the conformational changes of the BH-H_C _are based on certain X-ray structures of bacterial RNAPs that were fortuitously crystallized in a kinked Bridge Helix conformation [5,8]. Kinking of BH-H_C _is stabilized by intramolecular interactions between amino acid side chains flanking each side of the hinge ([8,20]; Heindl *et al.*, unpublished observations). The location of the BH-H_C _and its kinking properties are thus pre-determined by the local amino acid sequence. This raises the question as to whether intrinsic structural features could also account for the molecular mechanism underlying BH-H_N _function. Inspection of the primary sequence surrounding BH-H_N_, coupled with insights obtained from molecular dynamic (MD) simulations [34-36], provide a plausible explanation for the structural basis of the BH-H_N _hinge. Systematic sampling of conformational states at five picoseconds (ps) intervals in a series of 27 independent 200 ps semi-quantitative MD simulations highlights the presence of distinct areas prone to local unfolding, with the most prominent peak centered around *mj*A' G810 (Figure 3A). Under these simulation conditions, most other regions of the Bridge Helix, including the N- and C-termini, maintain their α-helical conformations at all stages throughout the simulations. Closer inspection of the simulated structures formed by BH-H_N _kinking reveals a molecular switching mechanism that can be rationalized directly on the basis of the primary amino acid sequence and α-helical geometry (Figure 3B). The kinking of BH-H_N _critically involves the two glycine residues G809 and G810 that are located immediately C-terminal to M808 and are essentially invariant in all archaeal and eukaryotic RNAPIIs (Figure 1B). Glycine residues display low helix-forming propensity because their high conformational flexibility is entropically unfavourable within geometrically constrained α-helical structures [37,38]. This increased flexibility allows G809 and G810 to flip out of the α-helical conformation to create a flexible hinge (Figure 3B). The flipped conformation is then stabilized further through a variety of non-covalent interactions of M808 with R811 and E812 (Figure 3C). The kinking model is based on elementary structural and thermodynamic principles (as represented by the MD force field), but also strongly supported by the phenotypes of the G809 and G810 mutagenesis series (Figure 2A). In both positions, any residue other than glycine causes a severe reduction in the catalytic activity of RNAP due to an increased helix propensity, which reduces the likelihood of BH-H_N _kinking. In contrast, another glycine residue located slightly more C-terminal, G813, is noticeably less sensitive to change (Figure 2A). Similarly, the stabilization of BH-H_N _through van der Waals contacts between M808 and R811 and E812 appears to be relatively non-specific, so that a chemically diverse group of side chains are either acceptable (especially in the R811 position), or result in enhanced stabilization of the BH-H_N _kink (M808 substitution series). The fundamental requirement for a large side-chain in the M808 position is particularly evident from the fact that certain amino acids with smaller groups (G, A) are not capable of causing the superactivity associated with enhanced BH-H_N _kinking. It is therefore apparent that the molecular properties of BH-H_N_, such as its structural stability, are directly determined by the primary amino acid sequence and emerge spontaneously from MD simulations as a property inherent in the primary sequence of the Bridge Helix domain. Mutagenesis changes the biophysical properties of BH-H_N_, which is, in turn, directly reflected in altered NAC rates (Figure 2A).

### BH-H_N _and BH-H_C _operate in environments with widely different structural constraints

The presence of two distinct hinges in the Bridge Helix raises the question whether BH-H_N _and BH-H_C _are involved in the same mechanism during the NAC. Kinking of either of the two hinges will result in considerable local distortions, predicted to include a spatial redeployment of amino acid side chains and changes in the overall length, flexibility and general topology of the Bridge Helix domain. Kinking of BH-H_N _could result in altered interactions with adjacent domains, such as the βD-II, Link and F-Loop domains (Figure 1A; Additional files 1, 2 and 3), whereas hinge movements in BH-H_C _are expected to affect the position and/or mobility of the DNA-RNA hybrid and Trigger Loop conformation (Figure 1A; [20]).

**Figure 4:**
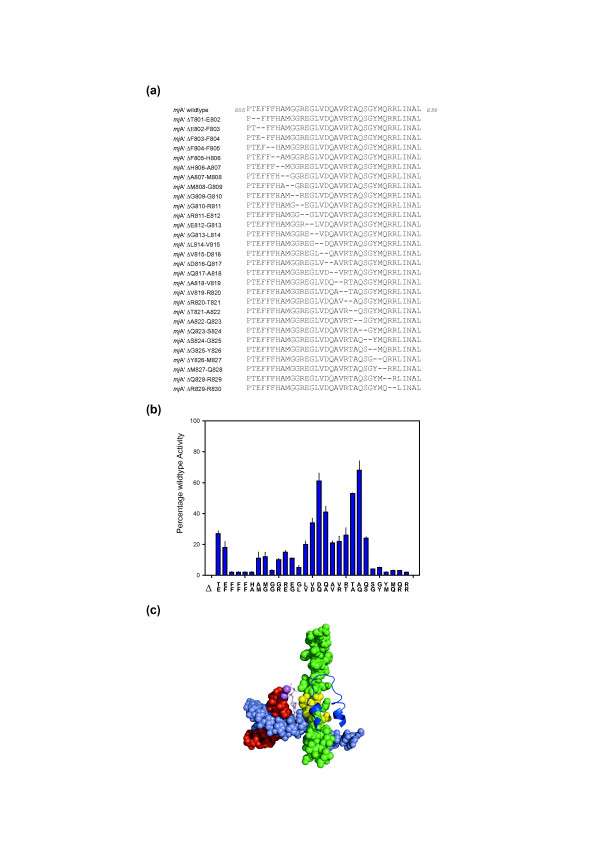
**Creation of locally twisted Bridge Helix structures**. **(A) **Sequences of the deletion constructs. A two-amino acid deletion window is moved systematically in a single residue step through the entire Bridge Helix primary sequence. The deletions remove two adjacent residues, but, more importantly, join the sequences bordering the mutation with 180° twist because of the removal of half an α-helical turn. **(B) **Activity of Bridge Helix mutants containing two-amino acid deletions shown relative to wildtype activity (100%). The amino acid pairs deleted from the primary sequence are shown vertically along the horizontal axis. Two distinct peaks of relative insensitivity to the deletions centered on *mj*A' ΔD816/Q817 and *mj*A' ΔA822/Q823 are discernible. All assays were performed in at least quadruplicate, with error bars showing standard deviation from the average value. **(C**) Position of the deletion-insensitive region of the Bridge Helix relative to other elements of the catalytic site. The Bridge Helix and other structures are shown using the same color-scheme as used in Figure 1A (yeast RNAPII elongation complex; [PDB #2E2H]). Residues orthologous to residues displaying the highest activity (>50%) levels in the two-amino acid scan (*mj*A' D816, Q817, T821, A822 and Q823) are highlighted in yellow.

Intriguingly, RNAP IV Bridge Helices also contain an additional two amino acid deletion, which would cause an even more radical change in the Bridge Helix by creating a local 180° twist of the α-helical structure (Figure 2C). This class of mutants combines strictly confined effects (removal of two adjacent side chains) with complex long range effects (realignment of remaining side chains on either side of the deletion to new positions and localized underwinding of the helical structure), which may possibly be coupled with the propagation of stress forces to the N- and C-terminal anchoring points of the Bridge Helix. The radical nature of such twisting mutations serves as an ideal tool to gain a deeper insight into structural constraints acting on the entire Bridge Helix. A series of two-amino acid deletions was prepared (Figure 4A). The results show that the BH-H_C _region is indeed remarkably resistant to such major conformational changes (Figure 4B). Although X-ray structures provide apparently persuasive arguments for certain residues of the Bridge Helix domain assisting in catalytic functions (for example, residues orthologous to T821 contacting the 3' end of the nascent transcript and/or the incoming rNTP [22]), the deletion phenotypes described here prove that such contributions are either redundant or non-existent in our archaeal system, which is highly conserved in this region. The results furthermore illustrate that, although BH-H_C _is located much closer than BH-H_N _to the substrates involved in the NAC, there are surprisingly few topological restraints. In marked contrast, none of the two-amino acid deletions support significant activity in the N-terminal part of the Bridge Helix (Figure 4B), proving that conformational changes are much more restricted in that region (which is consistent with the higher degree of evolutionary identity in the N-terminal half of the Bridge Helix compared to the C-terminal half; Figure 1). The results from the two amino acid deletion scan thus provide a first indication that the local conformational requirements for BH-H_N _and BH-H_C _differ quite radically.

**Figure 5:**
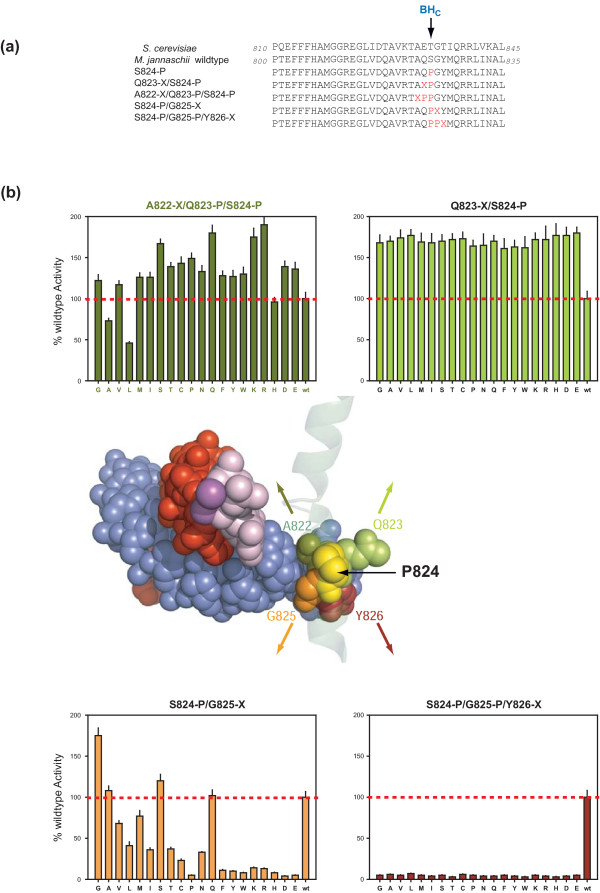
**Functional dissection of the BH-H_C _region**. **(A) **Position of mutagenized residues. The residues that differ from the archaeal wildtype sequence in the various mutagenesis constructs are shown in red. Wildtype sequences of the *M. jannaschii *and *S. cerevisiae *Bridge Helix sequences are shown for comparison. The position of BH-H_C _is marked with an arrow. **(B) **The center of the panel provides an overview depicting the orthologous residues in space-filling mode within the mutagenized part of the Bridge Helix modelled on the yeast RNAPII structure (PDB #2E2H); yeast residues E823 and T824 were replaced *in silico *with Q and P, respectively, to reveal the approximate location of these amino acids relative to the DNA-RNA hybrid and catalytic site). Adjacent parts of the Bridge Helix domain are shown as a transparent ribbon. All other colors are coded as in Figure 1A (template DNA is pale blue, the nascent transcript is red, the NTP in the insertion site is pink and catalytic metal ions are shown as magenta spheres). All mutants shown here contain the *mj*A' S824-P substitution (yellow). The results of promoter-independent activity assays are plotted relative to wildtype activity (indicated with a red dashed line). Two of the bar charts show the effect of introducing systematic substitutions in positions located immediately N- or C-terminal to S824-P (*mj*A' Q823 [lime green; upper right]; *mj*A' G825 [orange; lower left]). The other two bar charts show the functional consequences of introducing additional systematic substitutions in positions located immediately N-terminal to the double proline substitution Q823-P/S824-P (*mj*A' A822 [olive green; upper left]), or immediately C-terminal to the double proline substitution S824-P/G825 (*mj*A' Y826 [brown; lower left]). The colors of the histogram bars match the colors of the substituted residues in the structural model. All assays were performed in at least quadruplicate, with error bars showing standard deviation from the average value.

The substitutions *mj*A' M808-P and S824-P provide the strongest pieces of evidence for the existence and functional significance of BH-H_N _and BH-H_C_, respectively (Figure 2B[20]). Work in other systems has shown that the physicochemical properties of residues adjacent to prolines contribute considerably to the prevalence of *cis*/*trans *peptide bond isomers and therefore strongly influence kink geometry [39-42]. Additional proline substitutions of the positions immediately N- and C-terminal to either M808 or S824 (that is, *mj*A' A807-P/M808-P; M808-P/G809-P and Q823-P/S824-P; S824-P/G825) revealed examples of further intriguing differences between BH-H_N _and BH-H_C_: the double-proline substitution in BH-H_N _abolished the superactivity caused by M808-P, whereas the presence of an additional proline residue N-terminal to S824 continued to support the superactivity of S824-P (Figure 5B). The activity of the Q823-P/S824-P double mutant proves explicitly that the presence of two proline residues in this particular location of the Bridge Helix is not only compatible with catalytic function, but also compatible with superactivity. Prolyl-proline preferentially adopt an elongated polyproline II structure (87%), or less frequently (13%) a β-turn (Additional file 4A; [43,44]). Either of the structures would cause a substantial local increase in the flexibility of BH-H_C_.

In order to investigate this unexpected tolerance to the presence of two adjacent prolines in positions 823 and 824 in more detail, a complete substitution series of the residues around position *mj*A' S824-P was prepared, generating an assortment of systematic double-mutants (Figure 5A; *mj*A' Q823-X/S824-P and S824-P/G825-X; with X denoting 19 different variants). All substitutions N-terminal to S824-P (i.e. *mj*A' Q823-X/S824-P) displayed an almost completely invariant degree of extensive superactivity that was indistinguishable from the original S824-P mutant (Figure 5B). This result is remarkable because previous mutagenesis of Q823 revealed a broad spectrum of activity, ranging from substantial loss of function (Q823-C or I) to superactivity (Q823-D or E; [20]). It is evident that in the double-mutants the chemical nature of the side-chain residue in position 823 exerts no further functional influence, presumably because of the major distortion already caused by the proline substitution in position 824. Once such a gross structural alteration has occurred in S824-P, any additional changes in the adjacent N-terminal residue become structurally irrelevant.

Because Q823-P/S824-P displayed no loss of superactivity, the residue immediately N-terminal to the double-proline substitution was also permutated, resulting in variants containing three adjacent substitutions in the BH-H_C _hinge region (Figure 5A; *mj*A' A822-X/Q823-P/S824-P). The majority of these substitutions, including the triple proline mutant A822-P/Q823-P/S824-P (Figure 5C; Additional file 4B), still displayed clearly detectable superactivity, albeit at a slightly reduced level. The tolerance of the BH-H_C _hinge to radical mutagenesis, as previously observed in the two-amino acid deletion screen, is therefore also reflected in the unexpectedly high tolerance to multiple proline substitutions in that region. This geometric freedom is, however, also spatially limited: substitutions in positions C-terminal to S824-P (Figure 5A) were mostly inactive, indicating that despite the structural flexibility of the positions N-terminal to S824-P, the C-terminal positions are functionally constrained (Figure 5D; a similar trend is also apparent in the two amino acid deletion scan data [see Figure 4B]).

### BH-H_N _and BH-H_C _control activity of the catalytic site differently

**Figure 6:**
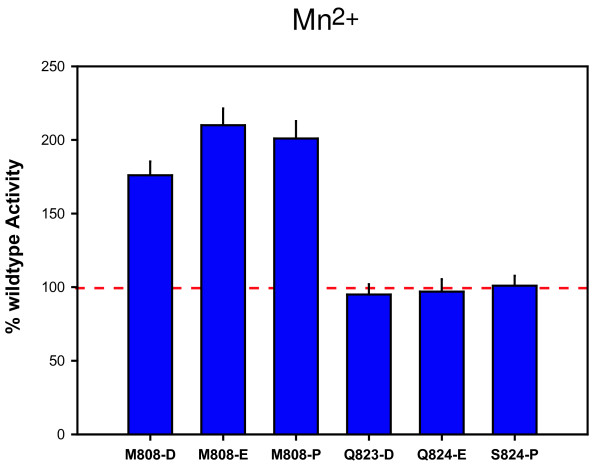
**Differential effect of Mn^2+ ^on superactivity in the BH-H_N _and BH-H_C _Region**. The activities of the most superactive substitutions in BH-H_N _and BH-H_C _are compared in Mn^2+ ^substituted reactions relative to the wildtype enzyme. The BH-H_N _substitutions (*mj*A' M808-D, -E and -P) continue to display a high degree of superactivity, whereas BH-H_C _substitutions (*mj*A' Q823-D, -E and S824-P) only transcribe at rates comparable to wildtype RNAP.

Taken together, the extensive body of mutagenesis data leads to the conclusion that the BH-H_N _and BH-H_C _structures operate within intramolecular environments that vary widely in their conformational constraints. The chemistry of the NAC is typically catalyzed by two magnesium ions, immobilized non-covalently within the active site (via the 'metal A' and 'metal B' motifs; for example, [45,46]). A complete substitution of Mg^2+ ^by Mn^2+ ^reduces the activity of archaeal RNAP to approximately 40% (data not shown); the reduced activity is probably caused by the slightly larger Mn^2+ ^ions causing suboptimal spacing [47], and Mn^2+^-catalyzed reactions being more promiscuous in their requirements for alignment of the reactive groups [48]. Intriguingly, assays of superactive BH-H_N _and BH-H_C _mutants in the presence of Mn^2+ ^demonstrated that they fell into distinct categories. *mj*A' M808-D, M808-E and M808-P continued to display superactivity, whereas the activities of S824-P or Q823-P/S824-P only reached wildtype levels under these conditions (Figure 6). We can therefore conclude that the superactive substitutions in the BH-H_N _and BH-H_C _regions have different consequences because conformational changes in different parts of the Bridge Helix affect separate stages of the NAC. The reduced catalytic activity caused by the presence of Mn^2+ ^ions in the active site becomes rate-limiting in Q823-D/E or S824-P, whereas M808-P overcomes this limitation to a large extent by stimulating transcription through an independent pathway, most likely involving the β-D II and Link domains.

## Discussion

### An expanded conformational repertoire of the Bridge Helix domain

The results presented here reveal several new surprising insights, including compelling evidence for the existence of a molecular hinge region in the N-terminal portion of the Bridge Helix and evidence for an unexpectedly large degree of tolerance to radical structural changes in the C-terminal part of this domain. It is apparent that the Bridge Helix domain displays a much greater conformational freedom than anticipated from currently available X-ray structures of RNAPs. Few, if any, of the residues of the Bridge Helix appear to make any specific contribution to catalysis other than through defining the nanomechanical properties intrinsic to the α-helical structure. The implications for mechanistic models aimed at describing the NAC are manifold, ranging from a re-evaluation of the structural basis of the RNAP translocation mechanism, to highlighting the hitherto neglected role of highly conserved domains in the catalytic site, and to obtaining a better understanding of the evolutionary diversity of Bridge Helices in different organisms.

Currently, we have only a limited understanding of the forces acting on the Bridge Helix that could drive localized conformational changes. Attempts to model the full NAC using molecular dynamics studies are severely limited by the large size of multi-subunit RNAPs and the immense computational effort that would be required to simulate the molecular events expected to last from 10s to 100s of milliseconds for the extension of a nascent transcript by a single nucleotide (approximately 30 ms/rNTP incorporation under optimal *in vivo *conditions; for example, [49]). The study of the intrinsic structural properties of individual domains by fully atomistic computer simulations reveal, however, interesting nanomechanical properties that have functional implications for the RNAP translocation mechanism [50-52]. The Bridge Helix domain contains intrinsically unstable α-helical regions that undergo spontaneous kinking motions, even in the absence of externally applied forces (Figure 3A). At least two of these unstable regions correspond precisely to the biochemically-mapped BH-H_N _and BH-H_C _regions. Strategically-placed glycine residues, such as *mj*A' G809, G810 (for BH-H_N_) and G825 (for BH-H_C_) provide the structural basis for forming these molecular hinges, with surrounding residues determining additional kinking parameters, such as the likelihood of kinking and/or the half-life of the kink after its isomerization (Figure 2A; Heindl *et al.*, unpublished observations). Interestingly, the simulations also highlight a potential third structurally labile region near the center of the Bridge Helix domain, spanning residues *mj*A' Q817 to R820. The relative sensitivity of this sequence to proline substitutions (Figure 2B) suggests, however, that this area of instability behaves functionally differently to BH-H_N _and BH-H_C_. It is possible that structural fluctuations in the central part of the Bridge Helix play a more dynamic role in supporting short-lived conformational changes that can either compensate for major structural rearrangements due to BH-H_N _and BH-H_C _kinking, or act as a store of 'fast' motions to lubricate the kinking of the hinge regions kinetically [34]. Although such hypotheses are currently beyond experimental proof, it is interesting to note that several, structurally unexplained superactivity mutants map to this central sequence (for example, *mj*A' D816-N; Q817-S/T/C/K and V819-K;Figure 2A[20]), and that this region is also exceptionally tolerant to radical twisting of the α-helical axis induced by deletions of two-amino acid segments (Figure 4B). Furthermore, data from anisotropic network mode analysis suggests that rigid body movements of the clamp domain may exert forces onto the center of the Bridge Helix via the Switch domains, potentially linking transcription to a ratchet-like translocation mechanism (Additional file 5; [53]). The Bridge Helix thus appears to have evolved specific nanomechanical properties that result in the controlled and highly localized isomerization of its conformation in response to allosteric alterations in the surrounding protein domains and nucleic acid substrates.

The Bridge Helix N-terminus is tightly surrounded by other domains, such as the β-D loop II [54], the experimentally uncharacterized Link domain [25,55], and the F-Loop [26]. As evident from the exceptionally high degree of sequence conservation (Additional files 1A, 2A and 3A), each of these domains is likely to play key roles in the NAC. The β-D II domain is a loop-like structure that interacts extensively in a side-way interaction with the central part of the Bridge Helix, while simultaneously maintaining direct physical contact with the most recently incorporated nucleotide (i-1 position). The interaction between the β-D II domain and nascent transcript also creates an extended binding pocket for the rNTP (additional file 1B, C). Similarly, the highly conserved Link domain is strategically placed to interact with the Bridge Helix N-terminus, β-D II domain, nascent transcript (i-1 and i-2 positions) and the incoming rNTP (Additional file 2B). Finally, an N-terminal extension of the Bridge Helix, the F-Loop, forms an extensive cap-like structure that contacts the Link domain and the tip of the Trigger Loop (Additional file 3B; [26]). The differential response of superactive substitutions in BH-H_N _and BH-H_C _to the presence of Mn^2+ ^in the catalytic site supports the view that conformational changes in these regions cause a distinct effect in the catalytic site of RNAP. While the C-terminal Bridge Helix operates predominantly by influencing Trigger Loop conformation, kinking of the N-terminus via BH-H_N _most likely alters the positions and/or conformations of the β-D II and Link domains, which are in direct physical contact with the nucleotide and nucleic acid substrates.

### *In vivo *occurrence of proline-substituted Bridge Helix hinges

**Figure 7:**
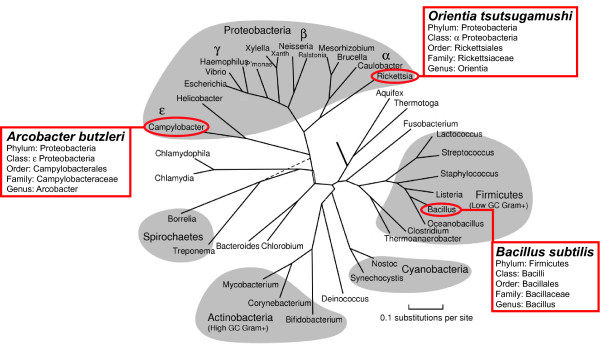
**Evolutionary positions of bacterial species with proline-containing Bridge Helices**. The evolutionary relationship between three bacterial species with proline-containing Bridge Helices is shown on a rooted phylogenetic tree calculated using maximum-likelihood methods from a concatenation of representative protein sequences [63]. The three species occupy widely divergent branches, strongly suggesting that the proline substitutions evolved independently, rather than were derived from a recently shared ancestor.

The existence and biological relevance of the experimentally determined hinge positions that tolerate proline substitutions is independently confirmed by a small number of naturally occurring Bridge Helix variants. Considering the large number of genomes sequenced thus far (currently including approximately 1,200 microbial and approximately 800 eukaryotic genomes; http://www.ncbi.nlm.nih.gov/sites/genome/), it can already be stated with confidence that naturally occurring proline substitutions are an exceedingly rare phenomenon. The three bacterial species that contain naturally occurring proline-substitutions in BH-H_N _represent two diverse bacterial phyla (Figure 7), and each of these phyla contains other closely related species with sequenced genomes, which do not contain any prolines in their Bridge Helices. Proline substitutions in the BH-H_N _region therefore appear to evolve spontaneously and independently in different bacterial lineages and subsequently remain restricted to individual species or strains. In at least some cases this evolutionary adaptation may be associated with significant simplifications of the transcriptional machinery, such as loss of the ω-RNAP subunit and absence of transcription-coupled repair (*O. tsutsugamushi*; [28,29]). These proline-containing Bridge Helix variants do not only provide strong and independent *in vivo *confirmation of the results identified in the high-throughput mutagenesis screen (Figure 2B), but prove that the results obtained in an archaeal model system also apply to the bacterial and eukaryotic domains; Bridge Helix kinking via two structurally independent molecular hinges is a universal mechanism operating across the entire evolutionary spectrum.

## Conclusions

The application of a high-throughput *in vitro *mutagenesis approach to the N-terminal portion of the *M. jannaschii *bridge helix domain has revealed a range of new insights that could not be anticipated from previously available structural and genetic data. The data sets (Figures 2A, 4B and 5B) clearly illustrate that many of the most interesting insights were derived from substitutions that would almost certainly not have been designed using a rational, structure-led approach (for example, *mj*A' M808-P; ΔD816/Q817; A822-P/Q823-P/S824-P). Furthermore, widely used methods, based predominantly on alanine-scanning mutagenesis [56], are also limited in their capacity to uncover some of the most interesting phenotypes (Figure 2A). It is therefore clear that automated high-throughput methods for generating site-directed mutants and assaying their phenotypic consequences will play an increasingly important role in exploratory investigations of protein structure/function relationships as part of a diverse strategy aimed at obtaining new insights into complex biological systems [57].

## Methods

### High-throughput mutagenesis

Combinatorial permutation libraries, containing all 19 variants with codon replacements optimized for expression in *E. coli *were purchased for *mj*A' H806, A807, M808, G809, G810, R811, E812, G813, Q823-X/S824-P, A822-X/Q823-P/S824-P, S824-P/G825-X, and S824-P/G825-P/Y826-X from GeneArt (Regensburg, Germany). The mutations, located within a BstBI-SbfI fragment of the codon-optimized C-terminus of the Bridge Helix [19] were transferred to a pET21a bacterial expression vector for the production of full-length, intein-free *mj*A' subunits. The presence of the desired mutations in the expression constructs was verified by DNA sequencing. DNA constructs containing the two amino-acid deletions across the Bridge Helix were purchased from GeneArt as synthetic gene fragments and transferred to bacterial expression plasmids as described above.

### RNAP Factory

The robotic procedures for high-throughput growth of bacterial expression strains, recombinant subunit purification and automated assembly into recombinant RNAPs (the 'RNAP Factory') have been described previously [19,20,58]. Briefly, bacterial constructs expressing the Bridge Helix mutants were transformed into chemically competent Acella cells (*ΔendAΔrecA *derivatives of *E. coli *BL21 [DE3]; EdgeBio, Gaithersburg, Maryland, USA). After growth for 16 to 18 hours at 37°C in 24-well plates in autoinduction medium (OverNight Express, Novagen, Nottingham, UK) the bacteria were harvested and used for a centrifugation-based robotic inclusion body purification protocol. The *mj*A' inclusion bodies were automatically solubilised in the presence of 8 M urea and quantitated at 562 nm with the bicinchoninic acid assay. Recombinant RNAPs containing the *mj*A' variants were assembled in a 96-well format dialysis cell using an urea-gradient from 6 M to urea-free spanning 16 hours at room temperature [19]. Each mutant subunit was expressed, purified and assembled *in vitro *at least in quadruplicate to assure consistency and reproducibility. The assembled RNAPs were harvested and used immediately for robotic transcription assays (see below). The assembly efficiencies of key mutants (including, among others, *mj*A' M808-P; A807-P/M808-P; M808-P/G809-P; S824-P; Q823-P/S824-P; S824-P/G825-P; A822-P/Q823-P/S824-P) were compared to assembly rates achieved with the wildtype *mj*A' subunit by assaying the reconstituted polymerases at limiting and saturating template DNA concentrations (see Additional file 19 in [20] for details and examples); no differences between wild-type and mutant enzymes were detected. For replacement of Mg^2+ ^ions in the catalytic site with Mn^2+ ^(for the transcription assays shown in Figure 6), the *in vitro *assembly process was carried out as described above, but with dialysis- and transcription buffers containing 10 mM Mn_2_-O-acetate instead of Mg_2_-O-acetate.

### *In vitro *transcription assays

The robotically implemented high-throughput trichloroacetic acid (TCA) precipitation assays, measuring the incorporation of (α-^32^P) rUTP into TCA-insoluble products, were carried out exactly as previously described [19,20]. Briefly, assay mixtures were incubated for 45 minutes at 70°C in thin-wall PCR plates before precipitating the radiolabeled transcripts by the addition of ice-cold TCA solution. After incubation for 30 minutes at 1°C, the nucleic acid precipitates were collected by vacuum filtration on a 96-GF/F glass fiber filter plate (Whatman, Maidstone, UK) and extensively washed with further aliquots of ice-cold TCA solution. After drying the filters, scintillant (MicroScint-O; Perkin-Elmer, Cambridge, UK) was added and the amount of incorporated (α-^32^P) rUTP quantified with a microplate counter (TopCount NXT, Packard, Cambridge, UK).

### High-throughput molecular dynamics simulation

Molecular dynamics (MD) simulations were performed using GROMACS [59]. In preparation for MD simulations, the archaeal Bridge Helix was modelled on the *S. cerevisiae *'active elongation' RNAPII structure (PDB #2E2H) using the SwissModel server in automated mode [60]. The simulation production runs were executed in a fully solvated atomistic production mode without restraints. The energies of the modelled structures were initially minimized in vacuum using GROMACS with an AMBER force field (http://ambermd.org/) on a CPU cluster of the National Grid Service (NGS). During pre-processing the system was warmed to 200K under the control of a Berendsen thermostat with a coupling constant of 1.0 ps. All structures were energy-minimized in pre-equilibrated simulation boxes filled with TP3 water, and sodium and chloride ions were added to a final concentration of approximately 150 mM. For production runs the temperature was increased to 300 K (27°C). The equations of motion were integrated using a step-size of two femtoseconds. The trajectories generated by 27 independent 200 picoseconds simulation runs, were analyzed using STRIDE [61], as implemented in VMD [62]. The frequencies of particular residues adopting a 'coil' conformation during 5 ps analysis windows were plotted relative to the Bridge Helix sequence.

## Abbreviations

BH-H_c_: molecular hinge located within the carboxy-terminal portion of the Bridge Helix; BH-H_N _: molecular hinge located within the amino-terminal portion of the Bridge Helix; NAC: nucleotide addition cycle; rNTP: ribonucleotide triphosphate; ps: picosecond; RNAP: RNA polymerase; TCA: trichloroacetic acid.

## Supplementary Material

Additional file 1 **Evolutionary conservation and structure of the β-D II domain**. **(A) **Alignment of β-D II domain sequences from bacteria (*E. coli *K12, *T. thermophilus*) and eukaryotes (*S. cerevisiae *and *H. sapiens*) against the archaeon *M. jannaschii*. Residues identical to the archaeal sequence are shown in red. The numbers flanking the sequences represent the location of the sequences within the open reading frame of the complete subunit. One of the residues in close contact with the nascent transcripts is boxed and identified with an arrow. **(B) **Arrangement of the β-D II domain relative to the RNAP active site. Most structures are shown in space-filling mode to emphasize spatial connections. The Bridge Helix is shown in green (with the BH-H_N _region (corresponding to *mj*A' M808-E812) highlighted in yellow), the β-D II domain in turquoise, the template DNA is pale blue, the RNA is red, the NTP in the insertion site shown as a pink stick model and catalytic metal ions as magenta spheres. The rNTP binding pocket is indicated with a white-dashed oval. **(C) **Close-up view of the β-D II domain. Note the extensive contacts between the β-D II domain with the rNTP and the i-1 position of the nascent transcript. Two potential β-D II/Bridge Helix contacts are mediated via residues orthologous to *mj*A' R811 and L814. ((PDB #2E2H); visualized with PyMOL). Click here for file

Additional file 2 **Evolutionary conservation and structure of the Link domain**. **(A) **Alignment of Link domain sequences from bacteria (*E. coli *K12, *T. thermophilus*) and eukaryotes (*S. cerevisiae *and *H. sapiens*) against the archaeon *M. jannaschii*. Residues identical to the archaeal sequence are shown in red. The numbers flanking the sequences represent the location of the sequences within the open reading frame of the complete subunit. Residues in close contact with the rNTP or nascent transcripts are indicated by boxes and arrows. **(B) **Arrangement of the Link domain relative to the RNAP active site. Most structures are shown in space-filling mode to emphasize spatial connections. The Bridge Helix is shown in green (with the BH-H_N _region (corresponding to *mj*A' M808-E812) highlighted in yellow), the Link domain in light purple, the template DNA is pale blue, the RNA is red, the NTP in the insertion site shown as a pink stick model and catalytic metal ions as magenta spheres. The rNTP binding pocket is indicated with a white-dashed oval. Click here for file

Additional file 3 **Evolutionary conservation and structure of the Link domain**. **(A) **Alignment of F-Loop domain sequences from bacteria (*E. coli *K12, *T. thermophilus*) and eukaryotes (*S. cerevisiae *and *H. sapiens*) against the archaeon *M. jannaschii*. Residues identical to the archaeal sequence are shown in red. The numbers flanking the sequences represent the location of the sequences within the open reading frame of the complete subunit. **(B) **Arrangement of the F-Loop domain relative to the RNAP active site. Most structures are shown in space-filling mode to emphasize spatial connections. The Bridge Helix is shown in green (with the BH-H_N _region (corresponding to *mj*A' M808-E812) highlighted in yellow), the F-Loop domain in lime, the template DNA is pale blue, the RNA is red, the NTP in the insertion site shown as a pink stick model and catalytic metal ions as magenta spheres. Click here for file

Additional file 4 **Analysis of proline conformational space in proteins**. (A) Conformation of peptide backbones containing a single (X-P-X; X is any other non-proline residue; left panel), two (X-P-P-X; central panel), or three subsequent proline residues (X-P-P-P-X) in protein structures displayed as Ramachandran plots (data generated using the web-based server described in [64]). The relative frequency of occurrence of particular φ/ω angle is encoded by the brightness of the square at the intersection of the coordinates. The φ/ω angle combination compatible with standard α-helical conformation is indicated with a dashed purple oval (left and central panels). Single proline residues conform to α-helical geometry when present at the extreme N- and C-termini of the α-helix, thus accounting for the occurrence of single prolines in the α-helical part of the plot in the left panel. For two or three adjacent proline residues, the only conformational space is in the top left quadrant of the plot, corresponding to polyproline-specific conformations. **(B) **Model of the extended poly-proline stretch in the *mj*A' A822-P/Q823-P/S824-P triple proline substitution mutant. The triple substitution mutant displays approximately 150% of activity in comparison to the wildtype enzyme (Figure 4B). The three proline substitutions are shown as yellow stick models and T821 is shown in red as a reference point pointing towards the catalytic site. The structure shown here was constructed using the *M. jannaschii *Bridge Helix sequence and conforms to the typical φ/ω angle combinations observed in α-helices and in polyproline structures. The structure is not necessarily an accurate model, but serves to demonstrate the increased local flexibility due to the presence of three subsequent proline residues. The model was created with Abalone http://www.biomolecular-modeling.com/Abalone/index.html. Click here for file

Additional file 5 **Gaussian Network Model Analysis**. The yeast RNAP elongation complex (PDB #2E2H) was subjected to Gaussian Network Model simulation (ignm.ccbb.pitt.edu; 6Å cut-off) to assess the distribution of forces within intact RNAPs. Slow-mode motions (rank 4, 6 and 8) affect particularly the center of the Bridge Helix (as indicated by green/gold color-coding), whereas the N- and C-terminal region remain immobile. The slow modes identify predominantly the response of individual domains to mechanical forces exerted on them from other structures during the simulation. It should be noted that such simulations do not take the chemical nature of residues into account and would therefore not be able to detect the intrinsic kinking properties of the BH-H_N _and the BH-H_C _regions. Click here for file
