# Supplementary figures and images for: The nucleotide addition cycle of RNA polymerase is controlled by two molecular hinges in the Bridge Helix domain
Source: BMC Biol. 2010 Oct 29;8:134. doi: 10.1186/1741-7007-8-134 (PMC2988716; doi:10.1186/1741-7007-8-134)

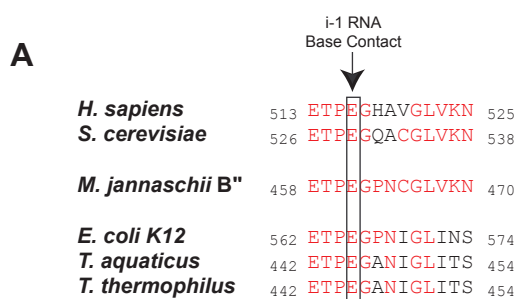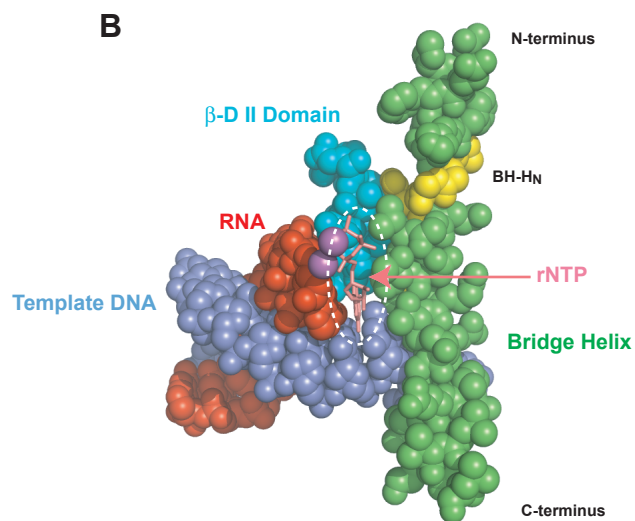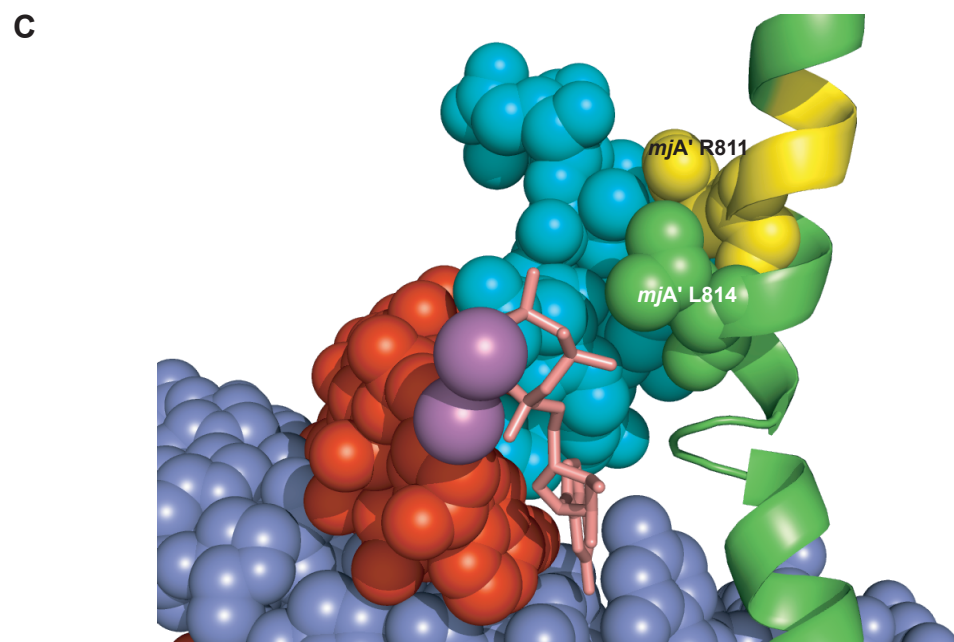

Supplement: Additional file 1 — Evolutionary conservation and structure of the β-D II domain. (A) Alignment of β-D II domain sequences from bacteria (E. coli K12, T. thermophilus) and eukaryotes (S. cerevisiae and H. sapiens) against the archaeon M. jannaschii. Residues identical to the archaeal sequence are shown in red. The numbers flanking the sequences represent the location of the sequences within the open reading frame of the complete subunit. One of the residues in close contact with the nascent transcripts is boxed and identified with an arrow. (B) Arrangement of the β-D II domain relative to the RNAP active site. Most structures are shown in space-filling mode to emphasize spatial connections. The Bridge Helix is shown in green (with the BH-HN region (corresponding to mjA' M808-E812) highlighted in yellow), the β-D II domain in turquoise, the template DNA is pale blue, the RNA is red, the NTP in the insertion site shown as a pink stick model and catalytic metal ions as magenta spheres. The rNTP binding pocket is indicated with a white-dashed oval. (C) Close-up view of the β-D II domain. Note the extensive contacts between the β-D II domain with the rNTP and the i-1 position of the nascent transcript. Two potential β-D II/Bridge Helix contacts are mediated via residues orthologous to mjA' R811 and L814. ((PDB #2E2H); visualized with PyMOL). [file 1741-7007-8-134-S1.PDF]

**A**

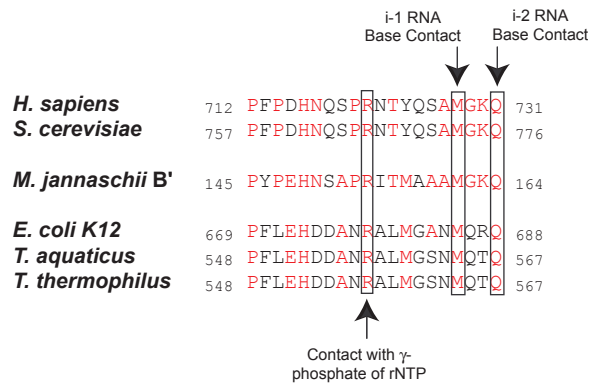

**B**

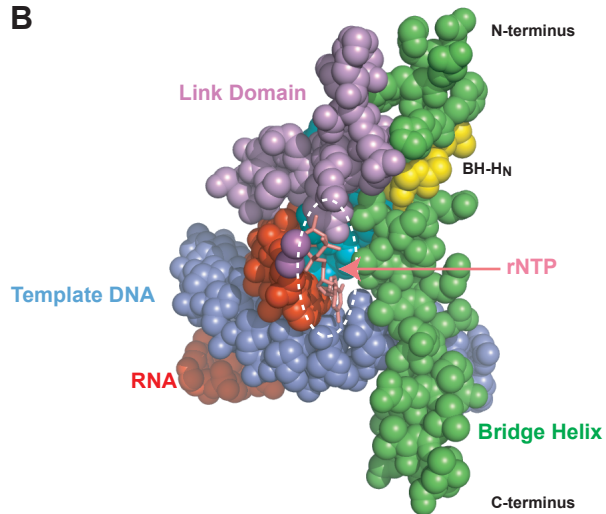

Supplement: Additional file 2 — Evolutionary conservation and structure of the Link domain. (A) Alignment of Link domain sequences from bacteria (E. coli K12, T. thermophilus) and eukaryotes (S. cerevisiae and H. sapiens) against the archaeon M. jannaschii. Residues identical to the archaeal sequence are shown in red. The numbers flanking the sequences represent the location of the sequences within the open reading frame of the complete subunit. Residues in close contact with the rNTP or nascent transcripts are indicated by boxes and arrows. (B) Arrangement of the Link domain relative to the RNAP active site. Most structures are shown in space-filling mode to emphasize spatial connections. The Bridge Helix is shown in green (with the BH-HN region (corresponding to mjA' M808-E812) highlighted in yellow), the Link domain in light purple, the template DNA is pale blue, the RNA is red, the NTP in the insertion site shown as a pink stick model and catalytic metal ions as magenta spheres. The rNTP binding pocket is indicated with a white-dashed oval. [file 1741-7007-8-134-S2.PDF]

A

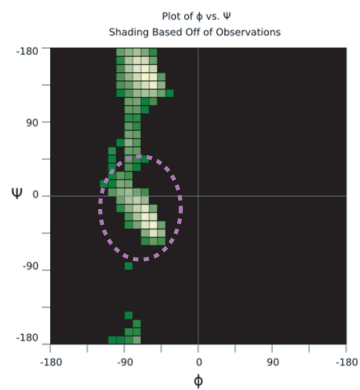

X-P-X

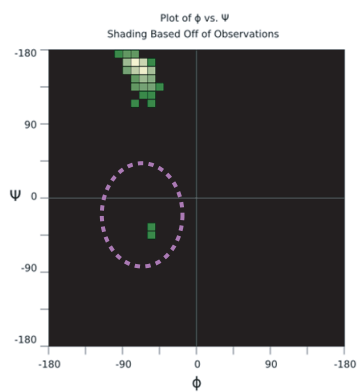

X-P-P-X

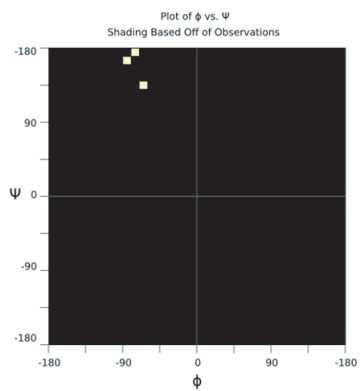

X-P-P-P-X

B

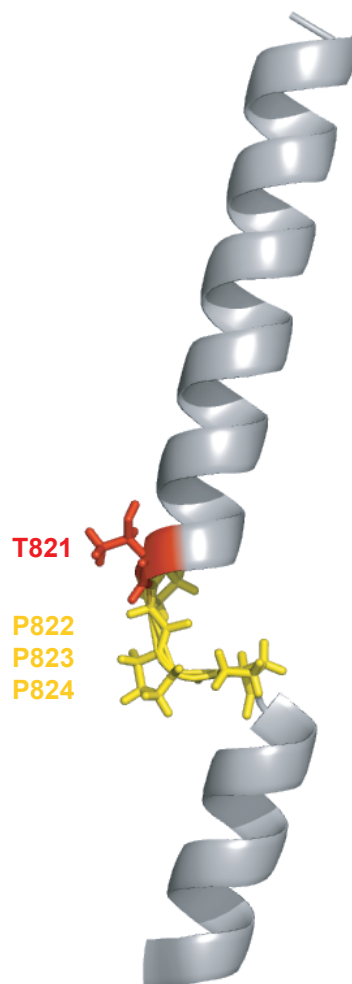

Supplement: Additional file 4 — Analysis of proline conformational space in proteins. (A) Conformation of peptide backbones containing a single (X-P-X; X is any other non-proline residue; left panel), two (X-P-P-X; central panel), or three subsequent proline residues (X-P-P-P-X) in protein structures displayed as Ramachandran plots (data generated using the web-based server described in [64]). The relative frequency of occurrence of particular φ/ω angle is encoded by the brightness of the square at the intersection of the coordinates. The φ/ω angle combination compatible with standard α-helical conformation is indicated with a dashed purple oval (left and central panels). Single proline residues conform to α-helical geometry when present at the extreme N- and C-termini of the α-helix, thus accounting for the occurrence of single prolines in the α-helical part of the plot in the left panel. For two or three adjacent proline residues, the only conformational space is in the top left quadrant of the plot, corresponding to polyproline-specific conformations. (B) Model of the extended poly-proline stretch in the mjA' A822-P/Q823-P/S824-P triple proline substitution mutant. The triple substitution mutant displays approximately 150% of activity in comparison to the wildtype enzyme (Figure 4B). The three proline substitutions are shown as yellow stick models and T821 is shown in red as a reference point pointing towards the catalytic site. The structure shown here was constructed using the M. jannaschii Bridge Helix sequence and conforms to the typical φ/ω angle combinations observed in α-helices and in polyproline structures. The structure is not necessarily an accurate model, but serves to demonstrate the increased local flexibility due to the presence of three subsequent proline residues. The model was created with Abalone http://www.biomolecular-modeling.com/Abalone/index.html. [file 1741-7007-8-134-S4.PDF]
