# Supplementary material for: The nucleotide addition cycle of RNA polymerase is controlled by two molecular hinges in the Bridge Helix domain
Source: BMC Biol. 2010 Oct 29;8:134. doi: 10.1186/1741-7007-8-134 (PMC2988716; doi:10.1186/1741-7007-8-134)

A

H. sapiens	769	MVVSGAKGSKINISQVIAVVGQQNVEGKRIPFGFKHRTLPHFIKDDYGPESRGFVENSYLAGLTP	833
S. cerevisiae	746	MVMAGSKGSFINIAQMSACVGQQSVEGKRIAFGFVDRTLPHFSKDDYSPESKGFVENSYLRGLTP	810
M. jannaschii A'	736	MAVTGARGNILNLTQMAACLGQQSVRGKRIFRGYRGRVLPHFEKGDLGARSHGFVRSSYKKGLSP	800
E. coli	725	MADSGARGSAAQIRQLAGMRGLMAKPDGSIIE------TP--------------ITANFREGLNV	769
T. aquaticus	1023	MAQSGARGNPQQIRQLCGMRGLMQKPSGETFE------VP--------------VRSSFREGLTV	1067
T. thermophilus	1023	MAQSGARGNPQQIRQLCGLRGLMQKPSGETFE------VP--------------VRSSFREGLTV	1067

B

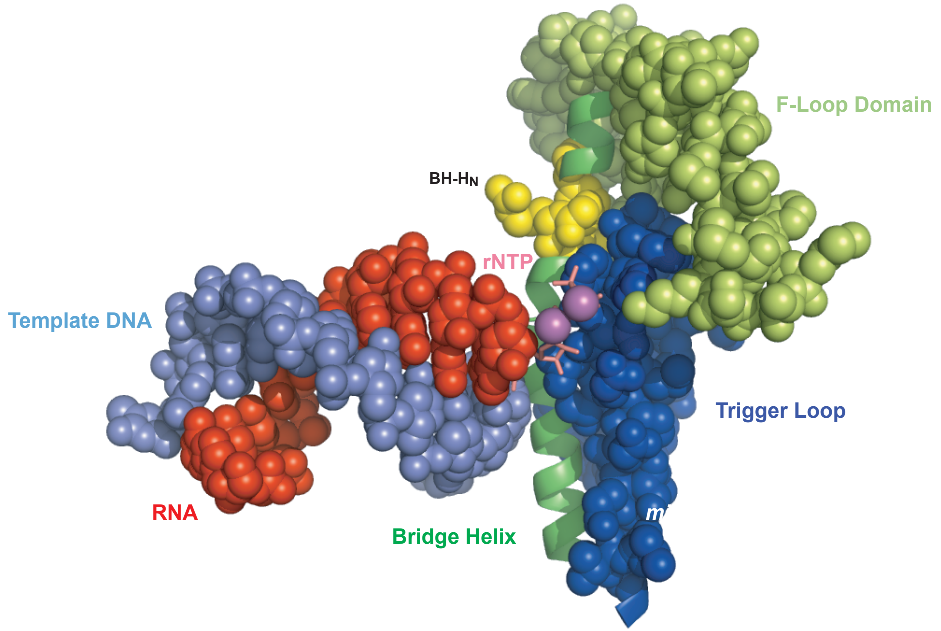

Supplement: Additional file 3 — Evolutionary conservation and structure of the Link domain. (A) Alignment of F-Loop domain sequences from bacteria (E. coli K12, T. thermophilus) and eukaryotes (S. cerevisiae and H. sapiens) against the archaeon M. jannaschii. Residues identical to the archaeal sequence are shown in red. The numbers flanking the sequences represent the location of the sequences within the open reading frame of the complete subunit. (B) Arrangement of the F-Loop domain relative to the RNAP active site. Most structures are shown in space-filling mode to emphasize spatial connections. The Bridge Helix is shown in green (with the BH-HN region (corresponding to mjA' M808-E812) highlighted in yellow), the F-Loop domain in lime, the template DNA is pale blue, the RNA is red, the NTP in the insertion site shown as a pink stick model and catalytic metal ions as magenta spheres. [file 1741-7007-8-134-S3.PDF]
