# Supplementary material for: The nucleotide addition cycle of RNA polymerase is controlled by two molecular hinges in the Bridge Helix domain
Source: BMC Biol. 2010 Oct 29;8:134. doi: 10.1186/1741-7007-8-134 (PMC2988716; doi:10.1186/1741-7007-8-134)
Supplement: Additional file 5 — Gaussian Network Model Analysis. The yeast RNAP elongation complex (PDB #2E2H) was subjected to Gaussian Network Model simulation (ignm.ccbb.pitt.edu; 6Å cut-off) to assess the distribution of forces within intact RNAPs. Slow-mode motions (rank 4, 6 and 8) affect particularly the center of the Bridge Helix (as indicated by green/gold color-coding), whereas the N- and C-terminal region remain immobile. The slow modes identify predominantly the response of individual domains to mechanical forces exerted on them from other structures during the simulation. It should be noted that such simulations do not take the chemical nature of residues into account and would therefore not be able to detect the intrinsic kinking properties of the BH-HN and the BH-HC regions. [file 1741-7007-8-134-S5.PDF]

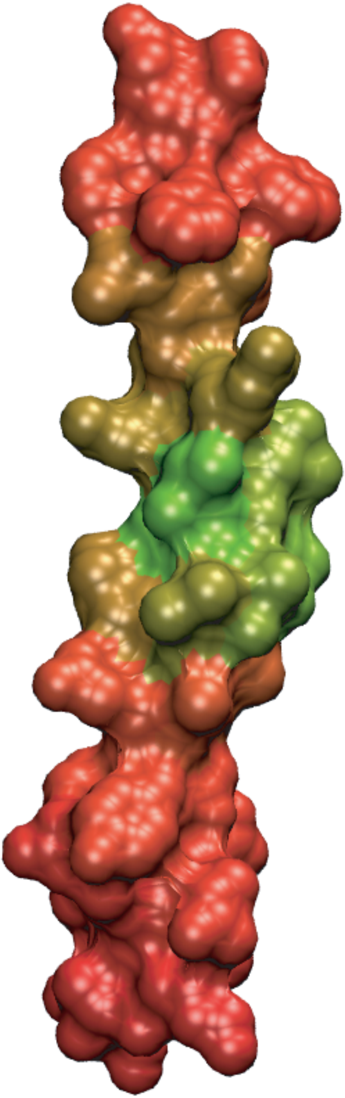

810 PRO
811 GLN
812 GLU
813 PHE
814 PHE
815 PHE
816 HIS
817 ALA
818 MET
819 GLY
820 GLY
821 ARG
822 GLU
823 GLY
824 LEU
825 ILE
826 ASP
827 THR
828 ALA
829 VAL
830 LYS
831 THR
832 ALA
833 GLU
834 THR
835 GLY
836 TYR
837 ILE
838 GLN
839 ARG
840 ARG
841 LEU
842 VAL
843 LYS
844 ALA
845 LEU
